# Trends and Outcomes of Hospitalized Influenza Patients With End-Stage Kidney Disease: Insights From the National Inpatient Sample 2010–2019

**DOI:** 10.7759/cureus.24484

**Published:** 2022-04-25

**Authors:** Guangchen Zou, Hongli Liu, Kaiqing Lin, Kaiwen Zhu, Tien-Chan Hsieh

**Affiliations:** 1 Internal Medicine, Danbury Hospital, Danbury, USA; 2 Internal Medicine, Rochester Regional Health, Rochester, USA

**Keywords:** in-hospital mortality, national inpatient sample, dialysis, end-stage kidney disease, influenza

## Abstract

Introduction

Influenza causes significant morbidity and mortality annually in the United States (US) and people with chronic medical conditions are thought to be at higher risk for severe disease and death. Infection is a leading cause of death for patients with end-stage kidney disease (ESKD). We used a national-level inpatient database to study the trend of influenza hospitalizations and in-hospital mortality for patients without and with ESKD.

Methods

The National Inpatient Sample (NIS) 2010-2019 was used. A primary diagnosis of influenza was identified using ICD-9-CM (487.X, 488.X) and ICD-10-CM codes (J09.X, J10.X, J11.X). ESKD was identified using a validated algorithm identifying patients with a diagnosis of ESKD or procedure code for dialysis and excluding patients with a diagnosis of acute kidney injury. Other diagnoses and procedures were identified using validated algorithms based on ICD-9-CM, ICD-10-CM, and ICD-10-PCS codes. Discharge-level weights were used to estimate the total number of admissions in the NIS universe. Weighted multivariable logistic regression was performed to study the association between ESKD and in-hospital death.

Results

131,942 admissions with a primary diagnosis of influenza with 4,647 admissions for ESKD patients among them were included in our analysis. Admissions varied by influenza season and ESKD patients accounted for 2.91% to 3.65% of all influenza admissions each season. 2,081 influenza patients (1.58%) died in the hospital and 115 patients with influenza and ESKD (2.47%) died in the hospital. Age-adjusted in-hospital mortality varied from season to season but was consistently higher in ESKD patients (2.25% vs 1.38%). ESKD was a risk factor for in-hospital death (OR 1.26, 95% CI 1.15-1.38) after adjusting for age, gender, primary payer, heart failure, chronic lung disease, obesity, drug abuse, immunocompromised status, bacterial pneumonia, the Charlson Comorbidity Index, and the influenza season.

Conclusion

ESKD patients accounted for a significant proportion of influenza hospitalizations in the US from 2010-11 to the 2018-19 influenza season. Among people hospitalized primarily for influenza, age-adjusted in-hospital mortality varied from season to season and was consistently higher in ESKD patients. For people hospitalized primarily for influenza, ESKD was an independent risk factor for in-hospital death.

## Introduction

Influenza causes significant mortality and healthcare resource utilization worldwide. In the United States (US), influenza caused 140,000 to 710,000 hospitalizations and 12,000 to 52,000 deaths annually from 2010 to 2020 according to estimates by the Center for Disease Control and Prevention (CDC) [1]. People with chronic medical conditions are thought to be at higher risk for more severe disease and death when they are infected with the influenza virus [2]. This study examines the trends of influenza hospitalization for all patients and patients with end-stage kidney disease (ESKD) and the in-hospital mortality for these patients from the 2010-2011 influenza season to the 2018-2019 influenza season using the National Inpatient Sample (NIS). The NIS was developed by the Agency for Healthcare Research and Quality (AHRQ). It is the largest publicly available all-payer inpatient healthcare database in the United States and contains data from more than 7 million hospital stays each year, and when weighted using discharge-level weight, can be used to estimate more than 35 million hospitalizations nationally [3]. This article was previously presented as a meeting abstract at the National Kidney Foundation Spring Clinical Meeting 2022 on April 7, 2022.

## Materials and methods

The NIS from the years 2010 to 2019 was used. Patients with a primary diagnosis of influenza were identified using the International Classification of Diseases, Ninth Revision, Clinical Modification (ICD-9-CM) codes (487.X, 488.X) and International Classification of Diseases, Tenth Revision, Clinical Modification (ICD-10-CM) codes (J09.X, J10.X, J11.X). Cases up to July were counted in the previous influenza season while cases starting in August were counted in the next influenza season. ESKD was identified using a previously validated algorithm which identified patients that (1) carried a diagnosis code for ESKD or (2) carried a procedure code for hemodialysis or peritoneal dialysis but (3) did not carry a diagnosis code of acute kidney injury [4]. Immunocompromised patients were identified using a previously validated algorithm identifying patients with organ transplants, HIV, malignancies, certain rheumatologic disorders, and other immunodeficiencies using ICD-9-CM and ICD-10-CM codes [5]. Individual diagnosis categories of the Charlson Comorbidity Index were identified, and the index was calculated using an algorithm developed by Deyo and colleagues and modified by Quan and colleagues using codes from the Manitoba Centre for Health Policy [6-8]. Bacterial pneumonia was identified using ICD-9-CM codes (481.X, 482.X) and ICD-10-CM codes (J13.X, J14.X , J15.X) [9]. Invasive mechanical ventilation was identified using a validated algorithm using ICD-9-CM codes and ICD-10-CM Procedure Coding System (ICD-10-PCS) codes [10]. Discharge-level weights provided in the NIS were used to estimate the total number of admissions for patients hospitalized with a primary diagnosis of influenza and the ESKD patients among them in the NIS universe, and the numbers were compared to the estimated number of all influenza-related hospitalizations for each influenza season from the CDC. In-hospital mortality rates were adjusted for age based on the age distribution of all admissions included in the analysis. Linear regression was performed to test the correlation between the estimated total number of influenza primary diagnosis admissions in the NIS universe and the CDC's estimated number of influenza-related admissions for each influenza season. Weighted multivariable logistic regression was performed to study the associations between ESKD and in-hospital mortality, using age, gender, primary payer, heart failure, chronic lung disease, obesity, drug abuse, and immunocompromised status, bacterial pneumonia, the Charlson Comorbidity Index, and the influenza season as covariates. Data processing was done using SAS® version 9.4 (SAS® Institute, Inc., Cary, NC). Some of the diagnosis and procedure codes used were listed in Table 3 in Appendices. Diagnosis and procedure codes used to identify the immunocompromised status and the Charlson Comorbidity Index can be found in the relevant references. Statistical analyses were performed using R version 4.0.3 (R Foundation). The study only used de-identified data from a public database and was exempt from IRB approval at both of our institutions.

## Results

**Figure 1:**
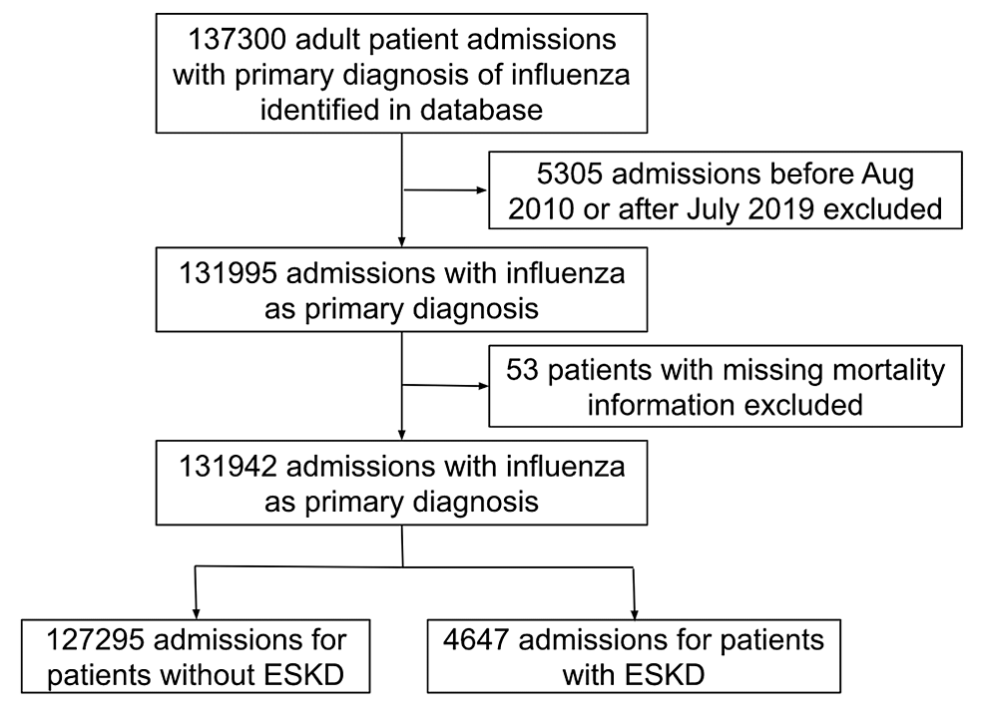
Study cohort

137,300 admissions for patients aged 18 or above were identified as having a primary diagnosis of influenza; 5,305 admissions before August 2010 or after July 2019 were removed and 53 were removed due to missing mortality data. 131,942 admissions were included in our analysis among which 4,647 were identified as admission for patients with ESKD (Figure 1).

Baseline characteristics of the admissions were listed in Table 1.

**Table 1:** Baseline characteristics of admissions included in the study. The admissions all carried a primary diagnosis of influenza and 4647 admissions were for patients with ESKD. IQR: interquartile range. SD: standard deviation.

		Not ESKD (n=127295)	ESKD (n=4647)	p value
Age group (%)	18-49	1731 (10.0)	82 (13.4)	<0.001
	50-65	3092 (17.8)	174 (28.3)	
	>65	12540 (72.2)	358 (58.3)	
Sex (%)	Male	55377 (43.5)	2322 (50.0)	<0.001
	Female	71910 (56.5)	2325 (50.0)	
Race (%)	Caucasian	88837 (73.0)	1882 (42.1)	<0.001
	African American	15357 (12.6)	1439 (32.2)	
	Hispanic	10827 (8.9)	689 (15.4)	
	Asian or Pacific Islander	2892 (2.4)	211 (4.7)	
	Native American	671 (0.6)	106 (2.4)	
	Other	3170 (2.6)	143 (3.2)	
Primary payer (%)	Medicare	85213 (67.0)	3818 (82.3)	<0.001
	Medicaid	11853 (9.3)	362 (7.8)	
	Private insurance	22872 (18.0)	369 (8.0)	
	Self-pay	4523 (3.6)	34 (0.7)	
	Other/no charge	2641 (2.1)	55 (1.2)	
All chronic lung diseaes (%)		54435 (42.8)	1635 (35.2)	<0.001
COPD (%)		35048 (27.5)	1052 (22.6)	<0.001
Immunocompromised status (%)		25531 (20.1)	1402 (30.2)	<0.001
Drug use (%)		3430 (2.7)	111 (2.4)	0.222
Heart failure (%)		26536 (20.8)	2026 (43.6)	<0.001
Diabetes without complications (%)		31907 (25.1)	1228 (26.4)	0.037
Diabetes with complications (%)		10467 (8.2)	2055 (44.2)	<0.001
HIV/AIDS (%)		599 (0.5)	54 (1.2)	<0.001
Charlson Comorbidity Index (mean (SD))		1.62 (1.31)	3.01 (1.26)	<0.001
Bacterial pneumonia (%)		8702 (6.8)	321 (6.9)	0.873
Invasive mechanical ventilation (%)		3216 (2.5)	138 (3.0)	0.066

**Figure 2:**
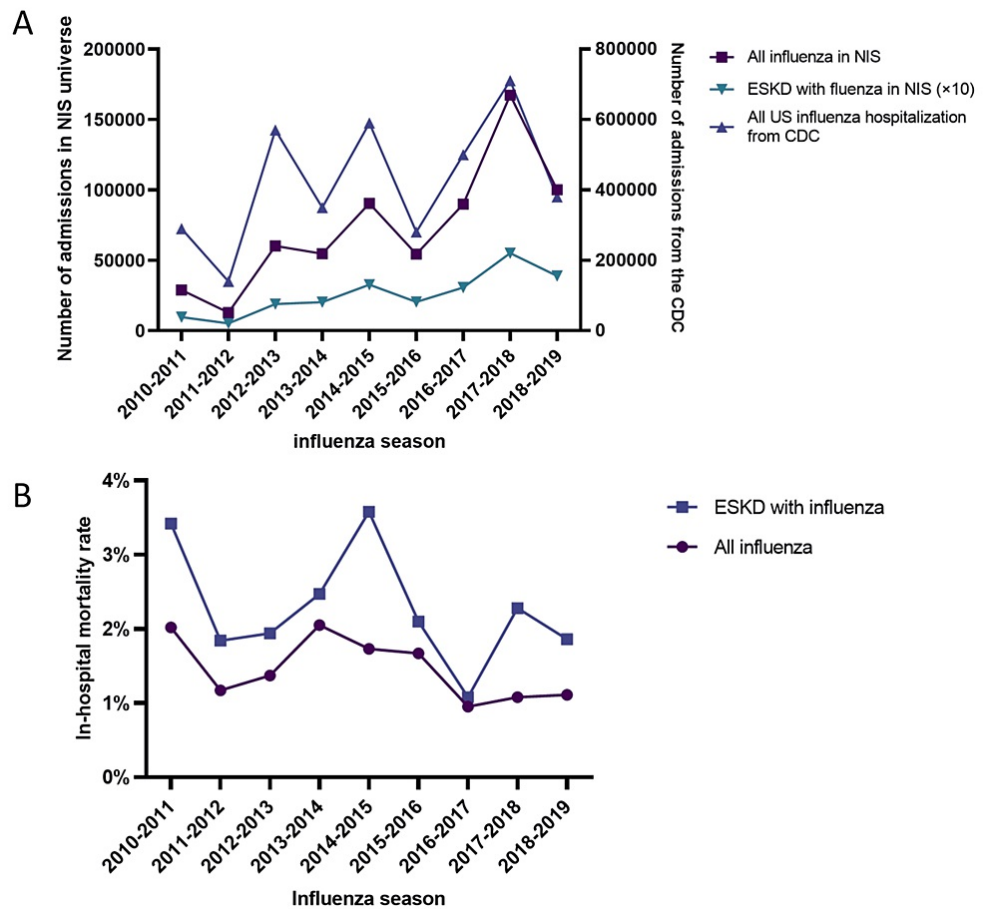
(A) Trends of estimated hospitalizations with a primary diagnosis of influenza and for ESKD patients with a primary diagnosis of influenza (magnified 10 times) in the NIS universe, compared with influenza-related hospitalizations in all of the US as estimated by the CDC for each influenza season from 2010 to 2019. Hospitalizations in the NIS universe were estimated using discharge-level weights provided in the NIS. (B) Age-adjusted in-hospital mortality for all admissions with a primary diagnosis of influenza and patients with ESKD and a primary diagnosis of influenza in the NIS for each influenza season from 2010 to 2019.

Using discharge-level weight provided in the NIS, the estimated number of hospital admissions with a primary diagnosis of influenza in the NIS universe varied from year to year and was the highest in the 2017-2018 influenza season. This followed the same trend as the total number of influenza-related hospitalizations in the US estimated by the CDC (linear regression R^2 ^= 0.69, p = 0.005). For each influenza season analyzed, ESKD patients accounted for 2.91% to 3.65% of all admissions with a primary diagnosis of influenza. (Table 2; Figure 2A).

**Table 2:** Estimated number of admissions with a primary diagnosis of influenza and estimated number of admissions for ESKD patients with a primary diagnosis of influenza in the NIS universe using discharge-level weights along with age-adjusted in-hospital mortality rate for both groups calculated using non-weighted data. CDC estimated influenza-related hospitalizations in all of the US were also included for comparison. *Data from the CDC.

Flu season	All admissions with primary influenza diagnosis in NIS		ESKD patients with primary influenza diagnosis in NIS			All influenza-related hospitalizations in the US estimated by the CDC*
	Number of admissions	In-hospital mortality (age-adjusted)	Number of admissions	In-hospital mortality (age-adjusted)		Number of admissions
2010-2011	28,861	2.02%	970		3.42%	290,000
2011-2012	12,888	1.17%	523		1.84%	140,000
2012-2013	60,170	1.37%	1,890		1.94%	570,000
2013-2014	54,620	2.05%	2,030		2.47%	350,000
2014-2015	90,435	1.73%	3,270		3.58%	590,000
2015-2016	54,410	1.67%	2,045		2.10%	280,000
2016-2017	89,885	0.95%	3,070		1.08%	500,000
2017-2018	167,355	1.08%	5,505		2.28%	710,000
2018-2019	100,185	1.11%	3,885		1.86%	380,000

**Figure 3:**
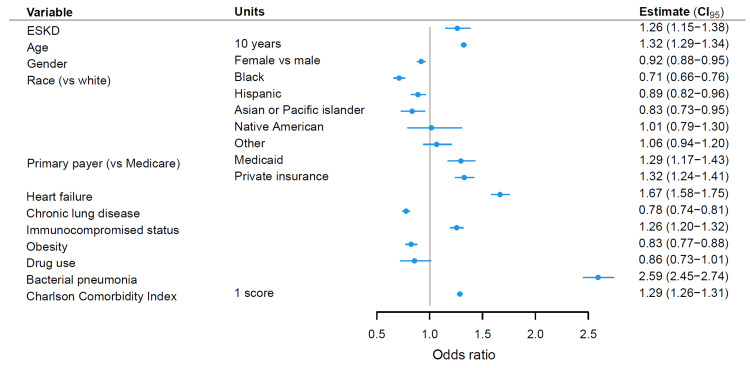
Forest plot of odds ratios from multivariable logistic regression analysis of in-hospital death using ESKD, age, gender, primary payer, heart failure, chronic lung disease, obesity, drug use, immunocompromised status, bacterial pneumonia, the Charlson Comorbidity Index, and the influenza season as variables and weighted for discharge weight. For patients hospitalized with a primary diagnosis of influenza, ESKD was associated with an odds ratio of 1.26 (95% CI 1.15-1.38) for in-hospital death adjusting for other factors. ESKD: end-stage kidney disease. CHF: congestive heart failure. CI: confidence interval.

Among the 131,492 admissions analyzed, 2,081 patients died in the hospital (1.58%). Among the 4,647 admissions where ESKD patients were hospitalized with a primary diagnosis of influenza, 115 died (2.47%). Age-adjusted in-hospital mortality was 1.38% for all influenza patients and 2.25% for patients with ESKD. The age-adjusted in-hospital mortality rate varied from influenza season to influenza season but was consistently higher in ESKD patients (Figure 2B). Among the influenza seasons analyzed, the age-adjusted in-hospital mortality rate was highest for ESKD patients in the 2010-11 (3.42% vs 2.02% for all patients) and the 2014-15 influenza season (3.58% vs 1.73% for all influenza patients). These two seasons were also among the seasons with the highest in-hospital mortality for all influenza patients (Table 2). The age-adjusted in-hospital mortality rate was the lowest in influenza season 2016-17 both for ESKD patients with influenza (1.08%) and all influenza patients (0.95%). In multivariable logistic regression, ESKD was associated with higher in-hospital mortality (OR 1.26, 95% CI 1.15-1.38) for patients hospitalized with a primary diagnosis of influenza after adjusting for age, gender, primary payer, heart failure, chronic lung disease, obesity, drug use, immunocompromised status, bacterial pneumonia, the Charlson Comorbidity Index, and the influenza season (Figure 3, Table 4 in Appendices). Other risk factors for in-hospital death include older age (OR 1.32, 95% CI 1.29-1.34 for every 10 years), heart failure (OR 1.67, 95% CI 1.58-1.75), immunocompromised status (OR 1.26, 95% CI 1.20-1.32), bacterial pneumonia (OR 2.59, 95% CI 2.45-2.74), and a higher Charlson comorbidity index (OR 1.29, 95% CI 1.26-1.31).

## Discussion

The study has important limitations. The NIS does not include admissions from the Veteran Affairs hospitals, Indian Health Service hospitals, and other federal hospitals. Certain data sources did not release discharge records for HIV/AIDS patients to the AHRQ [11], so the HIV/AIDS population may have been under-represented in the data. The diagnoses or procedures identified using diagnosis and procedure codes may have been affected by coding practices [12]. Important information such as medication use, and influenza vaccination status were not available. Other confounders may also have existed that were not adjusted for when analyzing in-hospital mortality. Furthermore, the different factors used in the analysis may not have been completely independent. Despite these, this large and relatively representative sample population gave us unique insights into the disease burden of influenza in ESKD patients and the higher risk of death ESKD patients faced when they were admitted for influenza.

Inpatient databases can be a valuable resource in studying influenza burden and outcomes. Although inpatient death from confirmed influenza cases grossly underestimated all influenza-related deaths, it has been shown to correlate very well with mortality burden estimated using the excess mortality method [13]. Similarly, in our study, although the number of estimated admissions with a primary diagnosis of influenza in the NIS universe is much lower than the number of CDC estimated national influenza-related admissions for each influenza season, the correlation was robust.

Of note, though the age-adjusted inpatient mortality rate was the lowest in the 2016-17 influenza season, admission numbers were actually relatively high (Figure 2). A similar pattern was found in the Society of Actuaries analysis of the 2016-17 influenza season using data from the CDC [14]. The reason for this was unclear. The variations in admission numbers and age-adjusted inpatient mortality from season to season were probably due to many factors including the characteristics of the predominant influenza strains of the season and the immunity level in the population.

The association between ESKD and higher in-hospital mortality is not surprising. Chronic kidney disease is associated with higher mortality for patients with respiratory tract infections [15] and infections of various kinds are a leading cause of death for patients with ESKD, accounting for up to 36% of all-cause mortality by some estimates [16,17]. In the US, seasonal variations in all-cause mortality of ESKD patients correlated with community influenza-like illness activity, suggesting more than 1,000 deaths in ESKD patients per year were potentially attributable to influenza-like illness [18].

Interestingly, in our model, certain comorbidities such as chronic lung disease were associated with a decreased risk of in-hospital death. A similar pattern has been found in other studies looking at risk factors for inpatient mortality of influenza or pneumonia using national inpatient registries [19,20]. One hypothesis is that this might be due to a lower threshold for admission in such patients resulting in patients with less severe influenza or pneumonia being admitted. People with high-risk medical conditions such as chronic lung disease were also more likely to be vaccinated [21], this could have potentially lowered their risk. 

Vaccination may potentially decrease the disease burden of influenza for ESKD patients. Though high-quality evidence is scarce on the effectiveness of influenza vaccination in the ESKD population, a pooled analysis of observational data suggested vaccination may protect against hospitalization and death in ESKD patients [22]. Influenza vaccination rates have improved over the years for ESKD patients in the US, but significant disparities exist among different dialysis facilities [23]. Providers should continue to make efforts to encourage ESKD patients to get influenza vaccines each season.

## Conclusions

ESKD patients accounted for 2.91% to 3.65% of hospitalizations with a primary diagnosis of influenza in the US from 2010-11 to the 2018-19 influenza season. The estimated number of admissions with a primary diagnosis of influenza in the NIS universe correlated with the CDC estimates of influenza-related hospitalizations during this period. Age-adjusted in-hospital mortality varied from influenza season to influenza season. Age-adjusted in-hospital mortality for EKSD patients hospitalized with a primary diagnosis of influenza during this time was consistently higher than that for all patients hospitalized primarily for influenza. For people hospitalized primarily for influenza, ESKD was an independent risk factor for in-hospital death (OR 1.26, 95% CI 1.15-1.38).

## Appendices

**Table 3:** Diagnosis and procedure codes used to identify some specific diagnoses and procedures. *Codes starting with.

Diagnosis/Procedure	ICD-10 CM/ICD-10 PCS codes	ICD-9 codes
End-stage kidney disease	Z992, N186, Z9115, Z4931, Z4901, Z4902, Z4931, Z4932	5856, 3995, 5498, V4511, V4512, V560, V561, V562, V5631, V5632, V568
Hemodialysis	5A1D*	3995
Peritoneal dialysis	3E1M39Z	5498
Acute kidney injury	N17*	584*
Bacterial pneumonia	J13*, J14*, J15*	481*, 482*
Invasive mechanical ventilation	5A1935Z, 5A1955Z, 5A1955Z, 0BH17EZ, 0BH18EZ	96.04, 96.05, 967*, 311*, 312*

**Table 4:** Results of multivariable logistic regression performed to study the association between ESKD and in-hospital death, using age, gender, primary payer, heart failure, chronic lung disease, obesity, drug use, immunocompromised status, bacterial pneumonia, the Charlson Comorbidity Index, and the influenza season as covariates and weighted for discharge-level weight. ^1^OR = Odds Ratio, CI = Confidence Interval

Characteristic	OR^1^	95% CI^1^	p-value
ESKD	1.26	1.15, 1.38	<0.001
Age (10 years)	1.32	1.29, 1.34	<0.001
Female gender	0.92	0.88, 0.95	<0.001
Race			
White	—	—	
Black	0.71	0.66, 0.76	<0.001
Hispanic	0.89	0.82, 0.96	0.002
Asian or Pacific islander	0.83	0.73, 0.95	0.007
Native American	1.01	0.78, 1.29	>0.9
Other	1.06	0.94, 1.20	0.3
Primary payer			
Medicare	—	—	
Medicaid	1.29	1.17, 1.42	<0.001
Private insurance	1.32	1.24, 1.41	<0.001
Self-pay	1.63	1.41, 1.87	<0.001
No charge	1.06	0.61, 1.71	0.8
Other	2.16	1.89, 2.46	<0.001
Heart failure	1.67	1.58, 1.75	<0.001
Chronic lung disease	0.78	0.74, 0.81	<0.001
Immunocompromised status	1.26	1.20, 1.32	<0.001
Obesity	0.83	0.77, 0.88	<0.001
Drug use	0.86	0.72, 1.01	0.067
Bacterial pneumonia	2.59	2.45, 2.74	<0.001
Charlson Comorbidity Index	1.29	1.26, 1.31	<0.001
Influenza season			
2010-11	—	—	
2011-12	0.55	0.46, 0.66	<0.001
2012-13	0.63	0.57, 0.70	<0.001
2013-14	0.94	0.85, 1.04	0.2
2014-15	0.69	0.63, 0.76	<0.001
2015-16	0.72	0.64, 0.80	<0.001
2016-17	0.38	0.34, 0.42	<0.001
2017-18	0.45	0.41, 0.49	<0.001
2018-19	0.44	0.39, 0.48	<0.001
